# Chart for Thermoelectric Systems Operation Based on a Ternary Diagram for Bithermal Systems

**DOI:** 10.3390/e20090666

**Published:** 2018-09-03

**Authors:** Julien Ramousse, Christophe Goupil

**Affiliations:** 1 Laboratoire Optimisation de la Conception et Ingénierie de l’Environnement (LOCIE), Université Savoie Mont Blanc, UMR 5271 Le Bourget du Lac, France; 2 Laboratoire Interdisciplinaire des Energies de Demain (LIED), Université Paris Diderot, UMR 8236 Paris, France

**Keywords:** finite time thermodynamics, ternary diagram for bithermal systems, operating modes, thermoelectric system optimal performance, figure of merit

## Abstract

Thermoelectric system’s operation needs careful attention to ensure optimal power conversion depending on the application aims. As a ternary diagram of bithermal systems allows a synthetic graphical analysis of the performance attainable by any work-heat conversion system, thermoelectric systems operation is plotted as a parametric curve function of the operating conditions (electric current and reservoirs’ temperature), based on the standard model of Ioffe. The threshold of each operating mode (heat engine, heat pump, thermal dissipation, and forced thermal transfer), along with the optimal efficiencies and powers of the heat pump and heat engine modes, are characterized graphically and analytically as a function of the material properties and the operating conditions. The sensibility of the performance aims (maximum efficiency vs. maximum power) with the operating conditions is, thus, highlighted. In addition, the specific contributions of each phenomenon involved in the semiconductor (reversible Seebeck effect, irreversible heat leakage by conduction and irreversible thermal dissipation by Joule effect) are discussed in terms of entropy generation. Finally, the impact of the exo-irreversibilities on the performance is analyzed by taking the external thermal resistances into account.

## 1. Introduction

Since thermoelectric phenomena were discovered at the end of 19th century, many works have been dedicated to the promotion of thermoelectric energy conversion. Such devices use the Seebeck (or Peltier) effect to directly convert heat into electricity (or inversely) [1] and, thus, belong to bithermal thermodynamic systems as they use two thermal reservoirs at distinct temperature levels to allow work-heat energy conversion. Although thermoelectric device applications remain limited due to their low energy conversion efficiency and to high semiconductor material cost, they show significant advantages compared with classical work-heat energy conversion systems: (i) Solid-state operation allowing maintenance-free operation without any moving parts and a long life-span of reliable operation; (ii) eco-friendly with no chemical reactions and gas-free emissions and (iii) large scalability and energy conversion reversibility (from heat into electricity and inversely). Consequently, extensive researches about the thermoelectric technology and its materials have been carried out in recent years for widespread application fields. Facing the various operating conditions aimed for a wide temperature range, a huge variety of semiconductor materials has been identified [2] and improving routes are still being explored for high-performance thermoelectric materials [3]. Particularly, highly efficient thermoelectric compositions including half-Heuslers and other classes as lead telluride and germanium telluride have been reported in References [4,5]. In addition, broad reviews of the main applicative fields and their respective challenges are given in Reference [6], with a focus on thermoelectric generators (TEG) in References [7,8] and thermoelectric coolers (TEC) in References [9,10]. With regard to TEG applications, a wide range of temperature and power levels is considered as this technology is explored for industrial processes, waste energy harvesting, solar power generation, the aerospace industry, combustion-driven vehicles, buildings, portable equipment, and electronics applications. The objectives are either to maximize the overall energy efficiency by cogeneration or to ensure electrical autonomy by eliminating the need for an external power source. Instrumentation applications need to be mentioned as thermoelectric effects are classically used for temperature (thermocouples) or heat flux measurements in TEG mode. A similar observation is made on the wide range of temperature and power levels considered for TEC applications, as they are investigated for industrial temperature control [11], medical applications [12], domestic air-conditioning systems for buildings, domestic and portable refrigerators, in the automobile industry, such as cooler/heaters in car seats and automobile air-conditioning applications, and for heat dissipation in electronic devices. Once again, the objectives (maximization of the Coefficient of Performance or of the thermal power) differ according to the application considered. In most of the cases, operating conditions are variable, thus, complicating the system design and control for optimal performance.

Facing the wide operating conditions encountered in these different applications, an overall description is needed to assist in their design and control to achieve high performance. Depending on the objective, some aim to maximize the useful power production, while others aim to increase the energy conversion efficiency. The various operating modes also call for a convenient analysis to evaluate the thermoelectric system’s potential as a function of the operating conditions.

A general description of thermoelectric bithermal system’s operation is discussed in Reference [13] which introduces a synthetic graphical representation of the different operating modes (heat engine, heat pump, or refrigeration system) of a thermoelectric material depending on the operating conditions (reservoirs’ temperatures and electrical current). However, some operating modes are not fully discussed, as discussed below. This paper aims at completing this description based on the ternary diagram representation for bithermal systems [14] along with analytical expressions of the optimal operating performance (maximum power vs. maximum energy conversion efficiency) and of the threshold of each operating mode. The entropy generation contributions, including endo-irreversibilities due to thermal conduction and Joule effect and exo-irreversibilities caused by the non-ideal coupling with the thermal reservoirs are also highlighted. Thus, this paper aims at providing a comprehensive and complete analysis of thermoelectric systems operation as a function of the operating conditions and the physical properties of the semiconductor material considered.

## 2. Materials and Methods 

### 2.1. Ternary Diagram of Bithermal Systems

**Figure 1:**
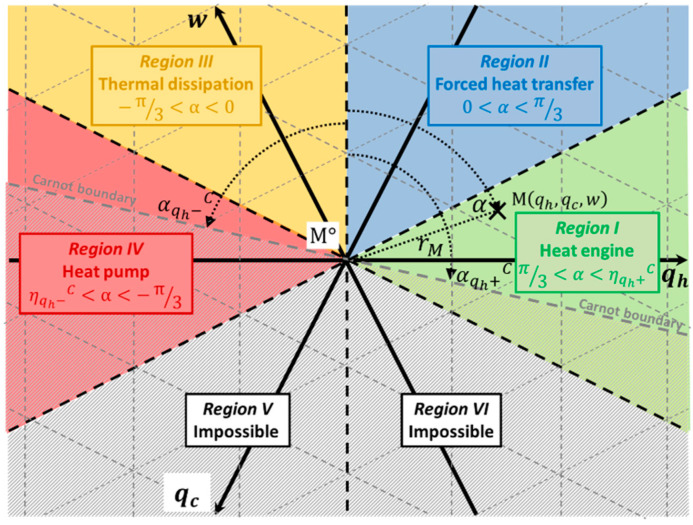
Ternary diagram of bithermal systems.

This paper aims to characterize the thermoelectric system’s operation on the ternary diagram $\left(q_{h},q_{c},w\right)$ for bithermal systems. This original and synthetic graphical representation has been presented in Reference [14]. Any bithermal thermodynamic system operating in cycles or steady state and exchanging heat with two distinct heat reservoirs at different temperatures to allow heat to work energy conversion can be plotted in the ternary diagram introduced in Figure 1. With respect to the first law of thermodynamics ($q_{h}+q_{c}+w=0$), the normed axes $q_{h}$, $q_{c}$ and w are linked by a 2*π*/3 in-plane rotation with the origin M° as the center of rotation. Thus, any bithermal system operation, characterized by the energy flows $q_{h}$, $q_{c}$ and w exchanged with its surrounding for given operating conditions, can be represented by a point $Μ\left(q_{h},q_{c},w\right)$ in the ternary diagram. The hot reservoir temperature is denoted *T_h_* and the cold one is *T_c_*, with $T_{h}>T_{c}$. The reservoirs’ temperature difference is denoted $\Delta T=T_{h}-T_{c}$ and the reservoirs’ temperature ratio θ=TcTh.

Depending on the sign of the energy flows $q_{h}$, $q_{c}$ and w (with respect to the conventional convention in mechanics, i.e., positive when entering the system and negative when leaving the system), six different regions of angle π3 (sextants) are clearly distinguishable, corresponding to different operating modes: (i) Heat engine (w<0, $q_{h}>0$, $q_{c}<0$); (ii) Forced heat transfer (w>0, $q_{h}>0$, $q_{c}<0$); (iii) Thermal dissipation (w>0, $q_{h}<0$, $q_{c}<0$) and (iv) Heat pump (w>0, $q_{h}<0$, $q_{c}>0$). Regions V (w<0, $q_{h}<0$, $q_{c}>0$) and VI (w<0, $q_{h}>0$, $q_{c}>0$) are not attainable with respect to the second law of thermodynamics. Regions I and IV stand for potentially reversible operating modes, whereas Regions II and III are dissipative operating modes.

Thanks to the normed axes, an intuitive graphical interpretation of the performance is derived by using polar coordinates. The energy flows intensity involved in the system is directly linked to its distance *r_M_* to the origin, and its efficiency is only related to the angle α. The radius *r_M_* expresses in a general way as the quadratic sum of the inlet and outlet energy flows involved in the system:

(1) $$r_{M}{}^{2}=e_{in}{}^{2}+e_{out}{}^{2}$$

where *e_in_/_out_* equals either the single energy flow involved in the direction considered or the energy flows difference divided by $\sqrt{3}$ when two energy flows are involved in the same direction. In other words, the radius is linked to the energy intensity required for the bithermal system operation. The higher the radius is, the greater the energy amounts exchanged by the system. Furthermore, the operating mode of the system considered is directly deduced from the angle *α*:

- α∈[π3;2π3]—Region I—Heat engine mode;
- α∈[0;π3]—Region II—Forced heat transfer mode;
- α∈[−π3;0]—Region III—Thermal dissipation mode;
- α∈[−2π3;−π3]—Region IV—Heat pump mode.

Furthermore, the system efficiency ($\eta_{q_{h}+}$ or $\eta_{q_{h}-}$) only depends on the angle α according to the following expression:

(2) $$\eta_{i}=\frac{1}{2}-\frac{\sqrt{3}}{2\tan\left|\alpha \right|}$$

with $\eta_{i}=\eta_{q_{h}+}=\frac{-w}{q_{h}}$ for α∈[0;2π3] and $\eta_{i}=\eta_{q_{h}-}=\frac{q_{c}}{-q_{h}}$ for α∈[−2π3;0].

Consequently, the higher the absolute value of the angle *α* is, the better the system efficiency (Equation (2)). 

Finally, the operation of bithermal systems is restricted to the top half-plane bounded by the Carnot boundary (for reversible systems), corresponding to a straight line passing through the origin Μ°(0,0,0) whose slope is a function of the reservoirs’ temperature ratio *θ*. The Carnot boundary is defined by the maximum angle $\alpha^{C}$ given by:

(3) $$\tan\alpha^{C}=\frac{\sqrt{3}}{2\theta -1}$$

where αqh+C∈[π3;2π3] and αqh−C∈[−2π3;−π3].

As a consequence, any real bithermal system must verify the condition $\left|\alpha \right|\leq \left|\alpha^{C}\right|$, and the entropy generation is directly linked to the difference between the angles α and $\alpha^{C}$, as:

(4) $$\frac{\sigma T_{c}}{q_{h}}=\frac{\sqrt{3}}{2}\left(\frac{1}{\tan\;\alpha }-\frac{1}{\tan\alpha^{C}}\right)$$

Thanks to this representation, any operating condition of a bithermal system can be synthetically interpreted graphically, with the help of polar coordinates. The authors thus propose to use the ternary diagram of bithermal systems to characterize thermoelectric systems operation for various material properties and operating conditions.

### 2.2. Thermoelectric Systems Model

Let us consider a bithermal thermoelectric system coupled to hot and cold reservoirs, at temperature *T_h_* and *T_c_*, respectively. Thermoelectric systems use the Seebeck (or Peltier) effect to directly convert heat into electricity (or inversely) and follow the classical theory of bithermal thermodynamic systems. Thermoelectric systems are composed of several legs, alternatively N- and P-type, connected in series electrically and in parallel thermally. The N-legs and P-legs are mostly made of bulk semiconductor materials (mainly Bismuth Telluride for working temperatures close to the ambient temperature). Since the thermoelectric power flows in steady state conditions are directly linked to the electric current fed to the system, we propose to report the performance of thermoelectric system as parametric curves in the ternary diagram for bithermal system (with the power flows ${\dot{Q}}_{h}$, ${\dot{Q}}_{c}$ and $\dot{W}$ as axes).

In a preliminary approach, thermoelectric elements are accurately described by the model proposed by Ioffe [15]. The validity of this simple approach has been validated in a previous study [16]. This model assumes a symmetrical distribution of the Joule effect between the hot and cold sides of the element and constant thermoelectric properties (the Seebeck coefficient *S*, the thermal conductivity λ and the electric conductivity *σ* estimated at the mean temperature *T_m_* of the hot and cold sides at *T_h_* and *T_c_*, respectively. As a consequence, the Thomson effect is neglected in this preliminary approach [16]. Further analysis of the influence of the Thomson effect on the performance is given in Reference [17]. For convenience, the temperature difference is denoted $\Delta T=T_{h}-T_{c}$. Based on the energy balance in the thermoelectric element, the thermal powers exchanged at the hot and cold sides of a thermo element of length *L* and section *A* are expressed (respecting the energy flow sign convention):

(5) Q˙c=SITc−RI22−KΔT

(6) Q˙h=−SITh−RI22+KΔT

where $\;R=\frac{\rho L}{A}$ is the electric resistance and $K=\frac{\lambda A}{L}$ is the thermal conductance.

Note that the Seebeck effect at the hot and cold sides could be either a heat pumping or a heat releasing effect, depending on the sign of the electric current. With respect to the first law of thermodynamics (${\dot{Q}}_{h}+{\dot{Q}}_{c}+\dot{W}=0$), the electric power is deduced from the hot and cold thermal powers:

(7) $$\dot{W}=-{\dot{Q}}_{c}-{\dot{Q}}_{h}=SI\Delta T+RI{}^{2}$$

The electric power is expressed as a quadratic function of the electric current. The voltage is, therefore, a linear function of the electric current:

(8) ΔV=W˙I=SΔT+RI

The external electric load, thus, writes:

(9) $$R_{load}=\frac{\Delta V}{I}=\frac{S\Delta T}{I}+R$$

To qualify the semiconductor thermoelectric performance, the material’s figure of merit is often used:

(10) $$Z=\frac{S{}^{2}}{\lambda \rho }$$

For convenience, the following notation is used to express the analytical optimal performance in Section 3.1.1 and Section 3.1.2:

(11) $$M=\sqrt{1+ZT_{m}}$$

According to the second law of thermodynamics ($\frac{{\dot{Q}}_{h}}{T_{h}}+\frac{{\dot{Q}}_{c}}{T_{c}}+{\dot{S}}_{gen}=0$), the entropy generation rate in the thermoelectric element volume is expressed as [18]:

(12) $${\dot{S}}_{gen}=\frac{RI^{2}}{2}\left(\frac{1}{T_{h}}+\frac{1}{T_{c}}\right)+K\Delta T\left(\frac{1}{T_{c}}-\frac{1}{T_{h}}\right)$$

From this expression, two contributions to the entropy generation are identified: Joule effect and thermal conduction. The Seebeck effect does not account for entropy generation as it is a fully reversible effect that converts heat into electricity (and inversely).

**Figure 2:**
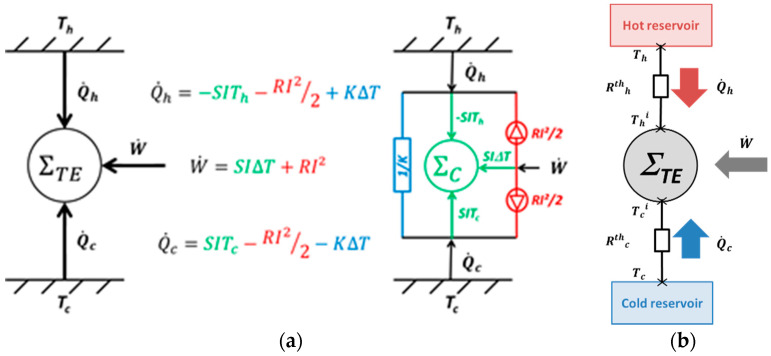
(**a**) Equivalent representation of thermoelectric systems without exo-irreversibilities; (**b**) Representation of the exo-irreversibilies via external thermal resistances.

Following, the thermoelectric system could be represented by the equivalent system shown in Figure 2a, by distinguishing a Carnot reversible system (Seebeck effect) with two irreversible contributions (thermal conduction and electric resistivity).

To complete the present analysis, exo-irreversibilities mostly encountered in practice, have also to be taken into account by adding external thermal resistances between the thermoelectric system and its thermal reservoirs, as shown in Figure 2b. The external thermal resistances modify the thermoelectric operating temperatures (denoted *T_c_^i^* and *T_h_^i^* in the following) as a function of the heats exchanged with the reservoirs:

(13) $${\dot{Q}}_{h}=\frac{\left(T_{h}-T_{h}{}^{i}\right)}{R^{th}{}_{h}},\;\mathrm{and}\;{\dot{Q}}_{c}=\frac{\left(T_{c}-T_{c}{}^{i}\right)}{R^{th}{}_{c}}\;$$

It results in additional irreversibilities increasing the entropy generation in the overall system. According to the second law of thermodynamics, the total entropy generation rate is then given by [18]:

(14) $${\dot{S}}_{gen}=\frac{RI^{2}}{2}\left(\frac{1}{T_{h}{}^{i}}+\frac{1}{T_{c}{}^{i}}\right)+K\Delta T^{i}\left(\frac{1}{T_{c}{}^{i}}-\frac{1}{T_{h}{}^{i}}\right)+\frac{\left(T_{h}-T_{h}{}^{i}\right){}^{2}}{R^{th}{}_{h}T_{h}T_{h}{}^{i}}+\frac{\left(T_{c}-T_{c}{}^{i}\right){}^{2}}{R^{th}{}_{c}T_{c}T_{c}{}^{i}}$$

In addition to thermal conduction and Joule effect, the two last entropy generation terms relate to thermal coupling between the thermoelectric system and its thermal reservoirs (on the hot and cold sides) which are a function of the heat flows exchanged. In case of null external thermal resistances, Equation (12) is retrieved as $T_{h}{}^{i}=T_{h}$ and $T_{c}{}^{i}=T_{c}$.

The thermoelectric properties and the geometry of the thermoelectric leg modeled are given in Table 1. Simulations are performed here for one thermoelectric leg for generalization purpose. In case of a thermoelectric module made of several legs, the energy flows have to be multiplied by the legs number associated thermally in parallel and electrically in series. Moreover, material properties are assumed constant as the mean temperature is kept set to 350 K. Furthermore, the temperature dependence of the material properties, and the consequent Thomson effect has been shown to have a slight influence on the thermoelectric performance [19].

## 3. Results and Discussion

Based on the thermoelectric model introduced in the previous section, a synthetic analytical and graphical analysis of the thermoelectric system performance is discussed in the following subsections with the help of ternary diagram representation for bithermal systems. Thanks to the model, the energy flows ${\dot{Q}}_{h}$, ${\dot{Q}}_{c}$ and $\dot{W}$ are set for given operating conditions (reservoirs’ temperature and electric current) and material’s geometry and properties, according to Equations (5)–(7). Thus, each operating condition considered corresponds to one operating point along the three axes ${\dot{Q}}_{h}$, ${\dot{Q}}_{c}$ and $\dot{W}$ of the ternary diagram. In addition, thermoelectric performance can be plotted in the ternary diagram as parametric curves function of the operating conditions (or material properties). The operating points could also be expressed as follows in the 2D orthonormal basis (${\vec{u}}_{q_{h}}$,${\vec{v}}_{q_{h}}$):

(15)  {x=Q˙h=−SITh−RI22+KΔTy=W˙−Q˙c3=SI(Th−2Tc)+32RI2+KΔT3 

First, a chart has been derived to highlight the influence of the operating conditions (reservoirs’ temperature and electric current) on the system operation. Analytical expressions for optimal performance leading either to maximum energy conversion efficiency or to maximum power are given, for both heat engine and heat pump modes. Then, the influence of the material properties is discussed with an emphasis on the entropy generation sources in the system. Finally, the impact of the exo-irreversibilities is discussed by considering additional thermal resistance between the thermoelectric system and its thermal reservoirs.

### 3.1. Influence of the Operating Conditions

**Figure 3:**
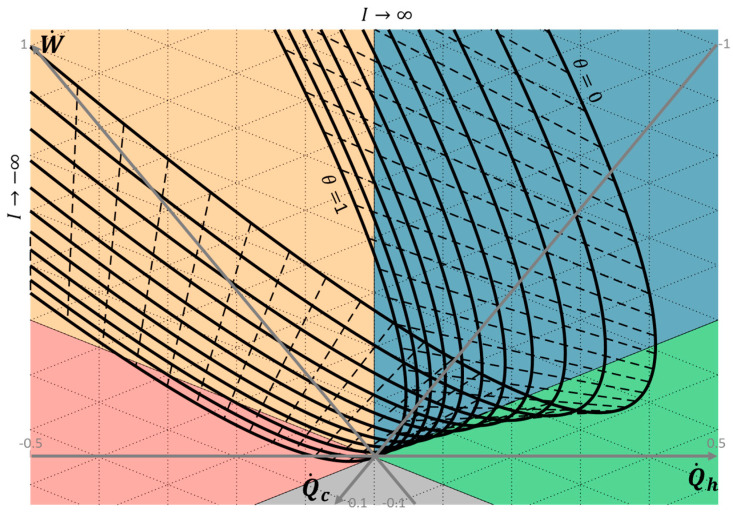
Chart of thermoelectric system operation as a function of the operating conditions (electric current *I* = [−4; 0.2; 4] and reservoirs’ temperature ratio *θ* = [0; 0.1; 1] with *T_m_*= 350 K). The grid step of the energy flows is 0.1 W. Solid lines: Constant reservoirs’ temperature ratio; dashed lines: Constant electric current.

This section is dedicated to highlighting the influence of the operating conditions (sources’ temperature and electric current) on the thermoelectric system’s operation. Figure 3 plots the operating points of the Bi_2_Te_3_ element detailed in Table 1, as parametric curves for the electric current *I* varying from −4 to 4 A with a step of 0.2 A and for reservoirs’ temperature ratio *θ* in the range 0 to 1 with a step of 0.1. The solid lines represent the system performance for one reservoirs’ temperature ratio value and varying electric current; the dashed lines relate to one electric current value and varying reservoirs’ temperature ratio.

The mean temperature *T_m_* is set to a constant 350 K to keep material properties constant, and the hot and cold reservoir temperature *T_h_* and *T_c_* are then deduced from the mean temperature *T_m_* and the reservoirs’ temperature ratio θ as:

(16) $$T_{h}=\frac{2T_{m}}{\theta +1}\;\mathrm{and}\;T_{c}=\frac{2\theta T_{m}}{\theta +1}\;$$

Figure 3 shows that the thermoelectric system operates in the different operating modes as previously described, depending on the operating conditions and the resulting signs of the energy flows. As detailed below, it can be observed that the thermoelectric system cannot operate in heat pump mode for low reservoirs’ temperature ratios (below 0.8, in Figure 3). The higher the reservoirs’ temperature ratio is, the higher the cooling power and the cold thermal energy conversion achievable. This result is consistent with the fact that a small temperature difference is required between the two sides of a heat pump to preserve a reasonable coefficient of performance. On the other hand, thermoelectric systems can always be operated in heat engine mode, for any reservoirs’ temperature ratio other than unity. The lower is the reservoirs’ temperature ratio, the higher the electric power and the electric energy efficiency. Figure 3 also highlights the influence of the reservoirs’ temperature on the ranges of the electric current applicable for each operating modes. Analytically, the electric current bounds of the different operating modes are simply given by solving:

(17) $$\dot{W}=0:\;I_{1,\dot{W}=0}=0\;\mathrm{and}\;I_{2,\dot{W}=0}=\frac{S\Delta T}{R}\;(i.e.,\;\Delta V=0)$$

(18) Q˙h=0: I1,Q˙h=0=SR(Th−Th2+2ΔTZ) and I2,Q˙h=0=SR(Th+Th2+2ΔTZ)

(19) Q˙c=0: I1,Q˙c=0=−SR(Tc+Tc2−2ΔTZ) and I2,Q˙c=0=−SR(Tc−Tc2−2ΔTZ) 

Note that the existence of roots for the cold heat flow is conditioned by:

(20) $$\frac{T_{c}{}^{2}}{\Delta T}>\frac{2}{Z}$$

Otherwise, the cold reservoir can only operate as a heat sink (${\dot{Q}}_{c}<0$) and the system cannot operate as a heat pump (with $COP_{c}=\frac{{\dot{Q}}_{c}}{\dot{W}}>0$ or $COP_{h}=\frac{-{\dot{Q}}_{h}}{\dot{W}}>1$). When the inequality of Equation (20) turns into equality, the maximum temperature difference allowing thermal pumping effect on the cold side is deduced for the material considered.

Following the parametric curves for given reservoirs’ temperature ratio in Figure 3, the thermoelectric system operates in the following operating modes, with increasing electric current:

- $I\in ]-\infty ;I_{1;{\dot{Q}}_{c}=0}]$ Thermal dissipation mode (Region III). The Joule effect dominates on both sides (high negative electric current), resulting in thermal dissipation of the electric power supplied at both thermal sinks.
- $I\in \left[I_{1;{\dot{Q}}_{c}=0};I_{2;{\dot{Q}}_{c}=0}\right]$ Heat pump mode (Region IV). The Seebeck effect on the cold side dominates the Joule effect and conduction (if Equation (20) is satisfied). Thus, thermal power is taken to the cold source to be transferred to the hot sink, with the electric power supplied.
- $I\in \left[I_{2;{\dot{Q}}_{c}=0};I_{1;{\dot{Q}}_{h}=0}\right]$ Thermal dissipation mode (Region III). The Seebeck and Joule effects dominate conduction on the hot side, resulting in thermal dissipation of the electric power at both sinks.
- $I\in \left[I_{1;{\dot{Q}}_{h}=0};I_{1;\dot{W}=0}\right]$ Forced heat transfer mode (Region II). The conduction effect dominates the Seebeck and Joule effects on the hot side (negative electric current). Thermal power is taken to the hot source to be transferred to the cold sink using the electric power supplied.
- $I\in \left[I_{1;\dot{W}=0};I_{2;\dot{W}=0}\right]$ Heat engine mode (Region I). The Seebeck effects on the hot and cold sides dominate the Joule effect, producing electric power from the thermal transfer transferred from the hot source to the cold sink.
- $I\in \left[I_{2;\dot{W}=0};I_{2;{\dot{Q}}_{h}=0}\right]$ Forced heat transfer mode (Region II). The Seebeck effect on the hot side and conduction dominate the Joule effect. As a result, thermal power is transferred from the hot source to the cold sink, using electric power.
- $I\in [I_{2;{\dot{Q}}_{h}=0};+\infty [$ Thermal dissipation mode (Region III), again. High electric current results in a high Joule effect on both sides that dissipates the electric power as thermal power to both heat sinks.

This representation completes that proposed in Reference [13], where PR and PC modes are defined with regards to the thermal power flow direction on the hot side. PR mode (meaning cooler) is defined for ${\dot{Q}}_{h}>0$ (hot heat source) and PC mode (i.e., heater) for ${\dot{Q}}_{h}<0$ (hot heat sink), with respect to the power flow convention adopted in this paper. The following operating modes description is proposed as equivalence:

- PR mode: Forced heat transfer mode (Region II).
- TG mode: Heat engine mode (Region I).
- PC mode (with  I<0), which covers the following operating modes: Forced heat transfer (Region II), thermal dissipation (Region III) and heat pump (Region IV) modes.

The representation proposed allows a more detailed description of the PC mode. The heat pump mode (with $COP_{c}>0$ or $COP_{h}>1$) operating conditions are identified, as well as for both dissipative operating modes (forced heat transfer and thermal dissipation modes). 

The maximum cooling effects at the cold reservoir (${\dot{Q}}_{c}>0$ maximum) and at the hot reservoir (${\dot{Q}}_{h}>0$ maximum), as well as the maximum work available ($\dot{W}<0$ minimum), are easily read on the graphs. The maximum efficiency is also directly deduced from graphs by the extreme values of the angle α. The following subsections focus on both heat engine and heat pump modes, with discussions on the optimal performance conditions.

The operating conditions are defined here with regards to the electric current applied to the thermoelectric system. However, as shown in Equation (8), the corresponding voltage also depends on the operating conditions. As a result, the external electric load has, thus, to be adapted to reach the operating point with respect to Equation (9), as it depends on the electric current (and the corresponding voltage) applied to the thermoelectric system. It has been shown that maximum energy conversion efficiency is reached with an adaptation of the external thermal and electric impedances to the internal impedances of the device [21].

As illustrated here, the ternary diagram allows a full analysis of the operating modes of thermoelectric systems based on graphical interpretation. As the influence of the reservoirs’ temperature and the electric current on the thermoelectric system is drawn, this chart may help to analyze the behavior of the system facing operating conditions variations (assuming internal steady state operation). Control command could thus be optimized with regards to the goal aimed.

#### 3.1.1. Focus on the Heat Engine Mode

**Figure 4:**
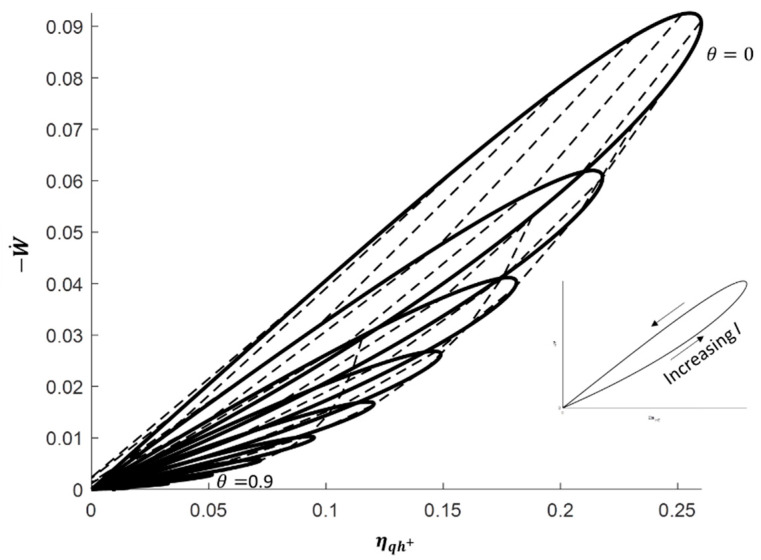
Electric power $-\dot{W}$ vs. electric energy conversion $\eta_{q_{h}+}$ for current *I* = [0; 0.2; 2.2] and reservoirs’ temperature ratio θ = [0; 0.1; 0.9] with *T_m_*= 350 K. Solid lines: Constant reservoirs’ temperature ratio; dashed lines: Constant electric current.

To deepen the discussion about the heat engine mode, the electric power produced is plotted against the energy conversion efficiency in Figure 4. The solid lines still represent the thermoelectric performance for one reservoir’s temperature ratio value and varying electric current *I* from 0 to 2.2 A; the dashed lines relates to one electric current value and varying reservoirs’ temperature ratio *θ* in the range 0 to 1.

The decrease of the maximum electric power and the maximum electric energy efficiency with the increase of the reservoirs’ temperature ratio is clearly highlighted. However, the high influence of the electric current is also emphasized. Although the optimal electric currents leading to maximizing either the electric power or the electric efficiency are close, distinct values are observed. This remark is confirmed by the analytical expressions given in Table 2, where the maximum electric power conversion is obtained by solving ∂(−W˙)∂I and the maximum conversion efficiency by solving ∂(ηqh+)∂I=0. These analytical expressions are in full agreement with those given in Reference [21]. It should be noted that, even if the optimal electric efficiencies only depend on the reservoirs’ temperature and the material figure of merit, the corresponding electric powers and optimal electric currents also depend on the thermal conductance and Seebeck coefficient of the material. Consequently, the knowledge of each material property (Seebeck coefficient *S*, thermal conductance *K* and electric resistance *R*) is needed to properly operate the thermoelectric system depending on the goal aimed.

#### 3.1.2. Focus on the Heat Pump Mode

**Figure 5:**
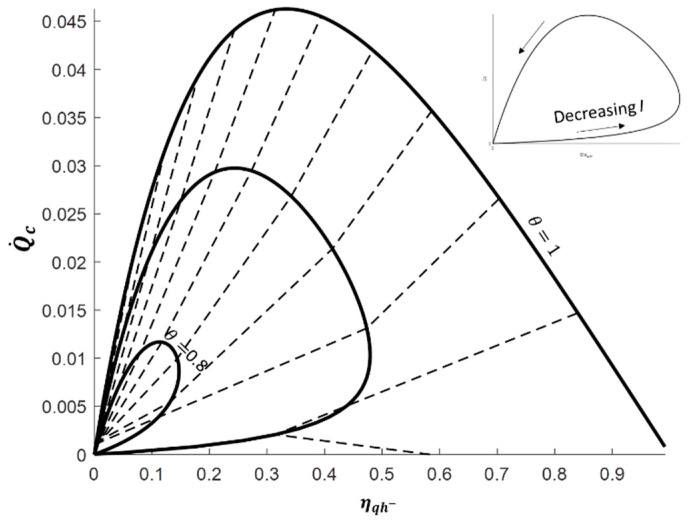
Cooling power $\;{\dot{Q}}_{c}$ vs. thermal energy conversion $\eta_{q_{h}-}$ for electric current *I* = [−1.6; 0.2; 0] and reservoirs’ temperature ratio *θ* = [0.8; 0.1; 1] with *T_m_* = 350 K. Solid lines: Constant reservoirs’ temperature ratio; dashed lines: Constant electric current.

Similar to the previous subsection, the cooling power of the cold reservoir is plotted against the energy conversion efficiency in Figure 5. The solid lines still represent the thermoelectric performance for one reservoir’s temperature ratio value and varying electric current *I* from −1.6 to 0 A; the dashed lines relate to one electric current value and varying reservoirs’ temperature ratio θ in the range 0.8 to 1. The reservoirs’ temperature ratio range is reduced due to the limitation mentioned in Equation (20).

This figure confirms the increase of the maximum cooling power and the maximum thermal energy efficiency with the increase of the reservoirs’ temperature ratio. However, the high influence of the electric current is also highlighted, leading to markedly distinct values of the optimal electric currents to maximize either the cooling power or the energy conversion efficiency. The analytical expressions of the optimal electric currents are given in Table 3, where the maximum cooling power is obtained by solving ∂(Q˙c)∂I, and the maximum conversion efficiency (maximum Coefficient of Performance) by solving ∂(ηqh−)∂I=0. Once again, although the optimal energy conversion efficiencies only depend on the reservoirs’ temperature and the material figure of merit, the corresponding cooling powers and optimal electric currents also depend on the material properties (Seebeck coefficient *S*, thermal conductance *K* and electric resistance *R*) that have to be known to operate the thermoelectric system with regards to the performance aimed.

### 3.2. Influence of the Endo-Irreversibilities 

**Figure 6:**
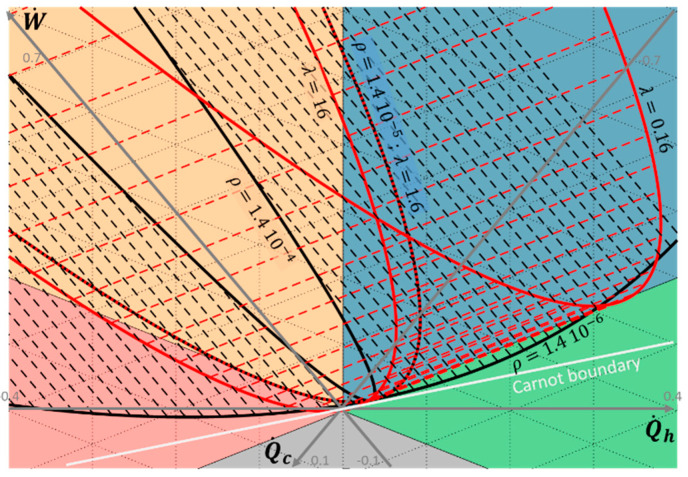
Chart of thermoelectric system operation as a function of the electric current *I* = [−5; 0.2; 5] and the material properties (*K* and *R*) for θ = 0.75 and *T_m_*= 350 K (*T_h_* = 400 K and *T_c_*= 300 K). Black solid lines relate to electric resistivity values *ρ* = [1.4 × 10^−6^; 1.4 × 10^−5^; 1.4 × 10^−4^] with *λ* = 1.6 W·m^−1^·K^−1^; Red solid lines relate to thermal conductivity values *λ* = [0.16; 1.6; 16] with *ρ* = 1.4 × 10^−5^ Ω·m; Dashed lines relate to constant electric current.

The influence of the material properties on the thermoelectric system performance and the resulting entropy generation is discussed in this subsection. To highlight both contributions in the total entropy generation, the thermal conductance *K* and the electric resistance *R* values are modified with a factor of 10 from the nominal value given in Table 1, for θ = 0.75 and *T_m_* = 350 K (*T_h_* = 400 K and *T_c_* = 300 K). The corresponding performance is plotted in the ternary diagram shown in Figure 6. The reservoirs’ temperature is chosen so that both heat pump and heat engine modes are attainable for nominal thermophysical properties given in Table 1. The influence of the Seebeck coefficient is not represented here, as it relates to reversible effect in the material. However, its influence could be deduced from the influence of the electric resistance as they are linked via the electric current (Figure 2a). Thus, an increase by a factor 10 of the electric resistance is equivalent to an increase of the equivalent electric current and of the Seebeck coefficient with a factor $\sqrt{10}$ (to keep the equivalent figure of merit, Equation (10)). 

Variation of the thermal conductance results in shifting the parametric curve along the $\dot{W}=0$ axis. Indeed, as shown in Figure 2a, thermal conductance only impacts the passive heat transfer, implying ${\dot{Q}}_{h}=-{\dot{Q}}_{c}$. It results in an increase of the maximum energy conversion efficiency (in both heat engine and heat pump modes) with a decrease of the thermal conductance. The maximum electric power remains unchanged, but the maximum cooling power is highly impacted up to become impossible for high values of thermal conductance.

Variation of the electric resistance results in highly influencing the performance of the thermoelectric system. Increasing the electric resistance results in decreasing the eccentricity of the hyperbola, while the point for I=0 (i.e., $\dot{W}=0$ and ${\dot{Q}}_{h}=-{\dot{Q}}_{c}$) is unchanged. Although the heat engine mode remains always attainable, the corresponding electric current range is strongly reduced with an increase of the electric resistance. On the other hand, the heat pump mode may not be any more possible for the high value of the electric resistance. In other words, the increase of the electric resistance reduces both the maximum useful powers ($-\dot{W}$ in heat engine mode and ${\dot{Q}}_{c}$ in heat pump mode) and maximum efficiencies in both heat engine and heat pump modes.

**Figure 7:**
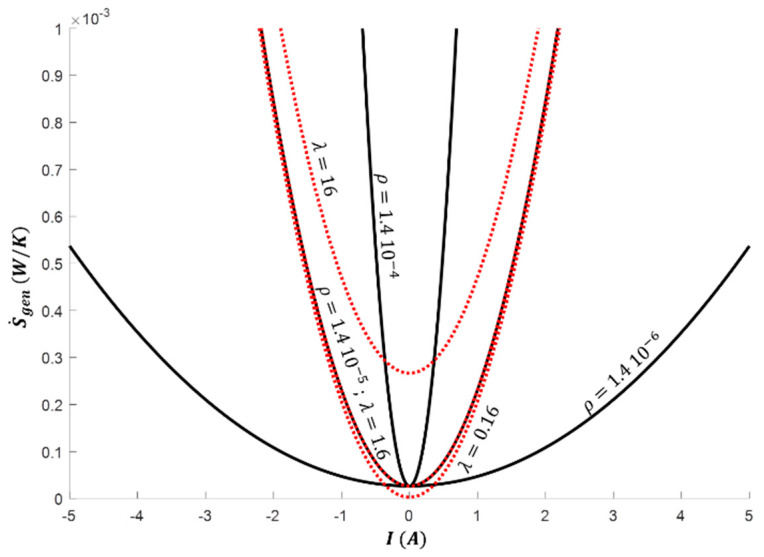
Total entropy generation ${\dot{S}}_{gen}$ vs. electric current *I* for *θ* = 0.75 and *T_m_*= 350 K (*T_h_*= 400 K and *T_c_* = 300 K). Black solid lines relate to electric resistivity values *ρ* = [1.4 × 10^−6^; 1.4 × 10^−5^; 1.4 × 10^−4^] with *λ* = 1.6 W·m^−1^·K^−1^; Red dashed lines relate to thermal conductivity values *λ* = [0.16; 1.6; 16] with *ρ* =1.4 10^−5^ Ω·m.

The total entropy generation (Equation (12)), including thermal conduction and electric resistance contributions, is plotted against the electric current for the previous material properties values in Figure 7. 

Symmetrical profile with the axis of symmetry I=0 results from the square electric current dependence of the entropy generation (Equation (12)). Thus, the higher the absolute value of the electric current is, the higher the entropy generation due to the electric resistance contribution. The entropy generation is then minimum for I=0, with non-null value due to the thermal conduction contribution. However, a low contribution of the thermal conduction to the total entropy generation is observed (corresponding to constant value deduced at I=0), due to the low temperature gradient in the operating conditions considered.

In case of null thermal conductivity, the hyperbola will shift to the left, along the $\dot{W}=0$ axis to pass through the origin *M*° for I=0. On the other hand, null electrical resistivity will convert the hyperbola to a straight line parallel to the Carnot boundary, passing to the point given for I=0, corresponding to passive heat transfer set by the thermal conductivity. In both cases, the figure of merit $ZT_{m}$ tends to an infinite value. However, both irreversible contributions (thermal conductivity *λ* and electric resistivity *ρ*) has to tend to null values to retrieve the ideal Carnot operation, implying $ZT_{m}\to \infty {}^{2}$. In addition, although the value of the figure of merit $ZT_{m}$ of semiconductor materials is a key parameter to estimate the optimal thermoelectric system efficiencies, this single parameter is not sufficient to characterize the thermoelectric system behavior: Each physical properties (*S*, *K* and *R*) has to be known independently.

Furthermore, it must be pointed out that the system performance sensibility to the operating conditions is a function of the material physical properties and dimensions (Figure 6 and Equations (17)–(19)). Consequently, the thermo element material and its sizing have to be carefully chosen as a function of the operating conditions range encountered in the application aimed. This observation is crucial for systems facing variable operating conditions, as illustrated in Reference [22] in case of a thermoelectric heat pump for building application with varying external temperature.

### 3.3. Influence of the Exo-Irreversibilities

**Figure 8:**
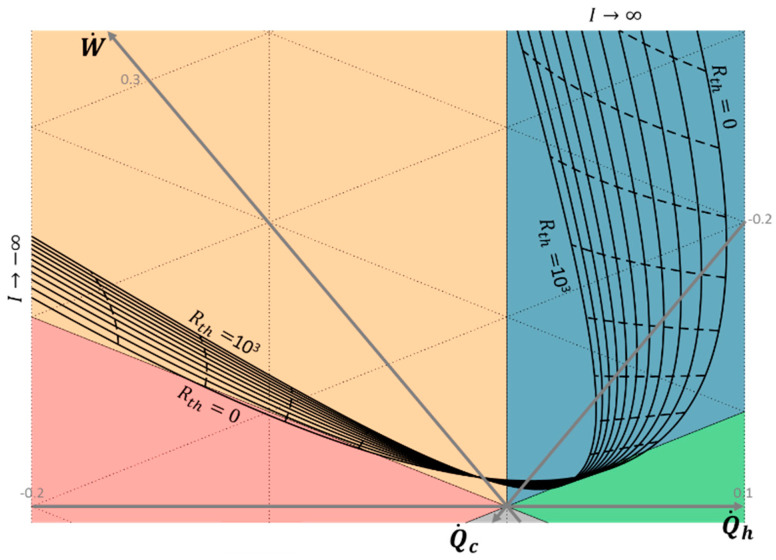
Chart of thermoelectric system operation as a function of the electric current *I* = [−5; 0.2; 5] and thermal resistances *R_th_*= [0; 100; 1000] for θ = 0.75 and *T_m_*= 350 K (*T_h_*= 400 K and *T_c_*= 300 K). Solid lines: Constant external thermal resistance; dashed lines: Constant electric current.

The non-ideal thermal coupling between the thermoelectric system and its reservoirs is taken into account by adding external thermal resistances, as shown in Figure 2b. Influences of the thermal coupling on the thermoelectric performance are discussed analytically in Reference [23]. To illustrate the impact of these exo-irreversibilities, Figure 8 plots the performance of the thermoelectric system for a hot reservoir at *T_h_*= 400 K and a cold reservoir at *T_c_*= 300 K (*T_m_*= 350 K and *θ* = 0.75), with thermal resistances varying from 0 to 10^3^ W/K, according to Equation (13). The solid lines relate to the thermoelectric performance for one external thermal resistance value and varying electric current from −5 to 5 A; the dashed lines relate to one electric current value and varying external thermal resistances.

As the thermal resistances directly influences the operating temperature of the thermoelectric system (Equation (13)), the energy flows exchanged are highly impacted. The increase of the external thermal resistances implies a decrease of the performances in both heat engine and heat pump modes. For the considered reservoirs’ temperature, the heat pump mode is no longer attainable as soon as the thermal resistance is higher than 60 W/K. Heat engine mode remains always possible, but the maximum electric power and the maximum electric efficiency drastically decrease with the increase of the external thermal resistances. The optimal performances given in Table 2 and Table 3 in heat engine and heat pump modes remain valid in the presence of external thermal resistances, by using the thermoelectric operating temperatures *T^i^* as a function of the heat flows exchanged with the thermal reservoirs (Equation (13)). Consequently, the operating electric current (or the corresponding electric load) has to be adjusted with regards to the external thermal resistances to reach the operating point aimed. Optimal energy conversion efficiency is reached when the external thermal and electric impedances are adapted to the internal impedances of the device [19].

These observations confirm the high impact of the thermal coupling between the thermoelectric device and its thermal reservoirs, that needs to be taken into account to reach the performance aimed.

## 4. Conclusions

Thanks to the ternary diagram for bithermal systems, a synthetic graphical and analytical analysis of the thermoelectric system’s performance is presented. This study focuses here on the Bismuth Telluride (Bi_2_Te_3_) material which is the most used thermoelectric material for working temperatures close to the ambient temperature. Obviously, this work may be revised for any thermoelectric material (such as Silicon-germanium SiGe or any other), resulting in similar tendencies. The influence of the operating conditions (reservoirs’ temperature and electric current) on the thermoelectric performance is highlighted and discussed. In addition to the potentially reversible operating modes (heat engine and heat pump modes) mostly studied, two dissipative operating modes can be encountered: Forced heat transfer and thermal dissipation modes. Thresholds of the different operating modes are identified, leading to highlighting the limit for heat pump mode operation. On the other hand, heat engine operation remains attainable whatever the reservoirs’ temperature and material properties, even with external thermal resistances, by adapting the operating electric current. Moreover, the optimal performance (maximum efficiency or maximum power) are deduced graphically and analytically. Finally, the entropy generation contributions (endo-irreversibilities due to thermal conduction and to electric resistivity, and exo-irreversibilities due to non-ideal thermal coupling to the reservoirs) are identified for further understanding. Consequently, this work allows a complete analysis of the thermoelectric performance with regards to the operating conditions. To conclude, this graphical analysis may be of high importance to define appropriate control of such system depending on the goal aimed, particularly in case of varying operating conditions.

As perspectives, the model could be improved by taking into account the temperature dependence of the material properties, thus, including the Thomson effect. However, a slight influence on the system performance is confirmed in the literature [20]. Furthermore, similar study has to be lead for classical thermodynamic cycles (Stirling, Erricson, Joule, etc.), to define synthetic charts for these bithermal systems as a function of the operating conditions and the physical properties of the fluid involved in the cycle. 

## Figures and Tables

**Table 1:** Geometric and thermophysical data for Bi_2_Te_3_ material at *T_m_* = 350 K [20].

Data	Value	Unit
Leg length, *L*	5 × 10^−3^	m
Leg section, *A*	1 × 10^−6^	m²
Seebeck coefficient, *S*	2.3 ×10^−4^	V·K^−1^
Thermal conductivity, *λ*	1.6	W·m^−1^·K^−1^
Electric resistivity, *ρ*	1.4 × 10^−5^	Ω·m
Figure of merit, *Z*	2.4 × 10^−3^	K^−1^

**Table 2:** Optimal performance in heat engine mode.

Operating Condition	$Max\left(\eta_{q_{h}+}\right)$	$Max\left(-\dot{W}\right)$
*I* [A]	$\frac{K\Delta T\left(M-1\right)}{ST_{m}}$	$\frac{ZK\Delta T}{2S}$
$-\dot{W}$ [W]	$\frac{K\Delta T{}^{2}}{T_{m}}\left[2+\frac{2}{ZT_{m}}-M\left(1+\frac{2}{ZT_{m}}\right)\right]$	$\frac{KZ\Delta T{}^{2}}{4}$
$\eta_{q_{h}+}$	$\left(1-\theta \right)\frac{M-1}{M+\theta }$	2ΔT3Th+Tc+8Z

**Table 3:** Optimal performance in heat pump mode.

Operating Condition	$Max\left(\eta_{q_{h}-}\right)$	$\;Max\left({\dot{Q}}_{c}\right)$
*I* [A]	$-\frac{K\Delta T}{ST_{m}}\left(1+M\right)$	$-\frac{ZKT_{c}}{S}$
${\dot{Q}}_{c}$ [W]	$\frac{K\Delta T}{T_{m}}\left[\left(T_{c}-\frac{\Delta T}{ZT_{m}}\right)\left(1+M\right)-T_{h}\right]$	$K\Delta T\left(\frac{ZT_{c}{}^{2}}{2\Delta T}-1\right)$
$\eta_{q_{h}-}$ [–]	$\frac{\theta M-1}{M-\theta }$	$\frac{ZT_{c}^{2}-2\Delta T}{ZT_{c}\left(2T_{h}+T_{c}\right)-2\Delta T}$

## Nomenclature

*A*	Thermoelectric leg section, m^2^
*COP*	Coefficient of performance, -
*I*	Electric current, A
*K*	Thermal conductance, W∙K^−1^
*L*	Thermoelectric leg length, m
*M*	Dimensionless thermoelectric factor, -
*q*	Specific thermal energy, J∙kg^−1^
$\dot{Q}$	Thermal power, W
*R*	Radius, J∙kg^−1^
*R*	Electrical resistance, Ω
*R_th_*	Thermalresistance, K∙W^−1^
*S*	Seebeck coefficient, V∙K^−1^
*S_gen_*	Entropy generation rate, W∙K^−1^
*T*	Temperature, K
*w*	Specific work, J∙kg^−1^
$\dot{W}$	Work power, W
*Z*	Figure of merit, K^−1^
**Greek Symbols**	
A	Angle, rad
∆*T*	Temperature difference, K
∆*V*	Voltage, V
*Θ*	Reservoirs temperature ratio, -
*H*	Efficiency, -
*Λ*	Thermal conductivity, W∙m^−1^∙K^−1^
*ρ*	Electrical resistivity, Ω∙m
**Superscripts and Subscripts**	
*c*	Cold reservoir
*C*	Carnot system
*h*	Hot reservoir
*load*	External load
*m*	Mean temperature
*q_c+_*	Hot heat source
*q_c−_*	Hot heat sink
